# Effect of early correction of hyponatremia on neurological outcome in traumatic brain injury patients

**DOI:** 10.1186/2197-425X-3-S1-A817

**Published:** 2015-10-01

**Authors:** K Ahmad, ZF Alrais, HM Elkholy

**Affiliations:** Critical Care Medicine, Rashid Hospital Dubai Health Authority, Dubai, United Arab Emirates

## Introduction

Hyponatremia is a most common electrolyte abnormality seen in Traumatic Brain Injury (TBI) patients. If it is not treated early it can lead to serious complications, affecting overall neurological outcome.

## Objectives

To know the effect of early correction of hyponatremia within 48 hours of onset on neurological outcome in TBI patients.

## Methods

A retrospective analysis of 150 cases admitted to Intensive Care Unit (ICU) diagnosed with different TBI who developed hyponatremia during their ICU stay, from January 2010 till October 2013. Neurological outcome was defined on the basis of initial Glascow Coma Scale (GCS) on admission to hospital and at the time of discharge from ICU. Good neurological outcome was defined if GCS improved significantly than admission; in severe TBI (if GCS 8 ≥3), in moderate TBI if ≥2, in mild TBI if ≥1. Poor outcome group defined, if GCS remained same or even deteriorated than admission. All patients were divided into two groups, whether serum sodium (S.Na) correction was achieved or not within 48 hours of onset of hyponatremia.Statistical association was seen between these two groups and neurological outcome groups. Data was analyzed by SPSS version 21.

## Results

87% were males & 13% female patients. Mean age was 24.43 years. 60%*(90/150)* cases were with severe TBI, and 29 & 31% were with moderate and mild TBI respectively depending upon their initial (GCS). Road traffic accident was the most common cause of TBI 46%*(70/150),* followed by fall from height, 41%. 13 cases due to industrial accidents, 3 sports related, 2 due to assault & 1 due to gunshot injury lead to TBI. In 81 patients (54%) different neurosurgical interventions (burr-hole,craniotomy, intracranial pressure monitoring-ICP and decompressive craniectomies) were performed depending upon the initial clinical condition. ICP monitoring was done in 47 cases as isolated or in combination with other therapeutic procedures. In 72% of cases correction of hyponatremia was achieved within 48 hours of onset and in 28% it took longer time (>48hours) to correct S.Na. Mean S.Na before treatment was 131.2+2.9, lowest value of 117 mmol/l and mean S.Na post treatment of hyponatremia was 137.6+2.5. Mean duration of onset of hyponatremia was 7.74 days after initial injury and ICU admission. Mean length of stay in ICU was 16.6+9.8, with minimum of 2 and maximum of 55 days .The highest length of stay was seen in severe TBI patients,18.3+8.9 days. Good neurological outcome group remained 83% *(125/ 150)* and poor outcome group 17%*(25/150).* In severe TBI (90/150), good neurological outcome was seen in 73 (81%) and poor outcome in 17 patients.There was statistically significant association seen between early serum sodium correction (within 48 hours of onset) and neurological outcome (Chi square with 1 degree of freedom = 19.286, P < 0.0001).

## Conclusions

There is significant association between early correction of hyponatremia and neurological outcome in traumatic brain injury patients.

**Figure 1:**
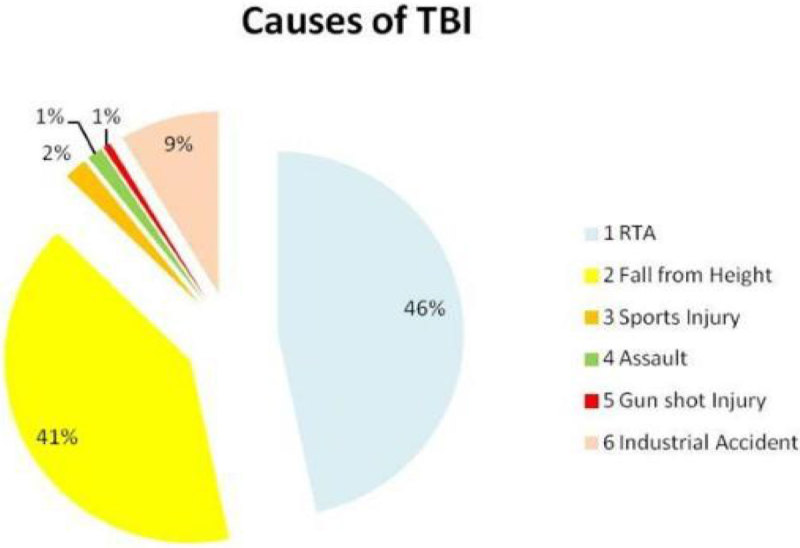
**[Causes of TBI].**

**Figure 2:**
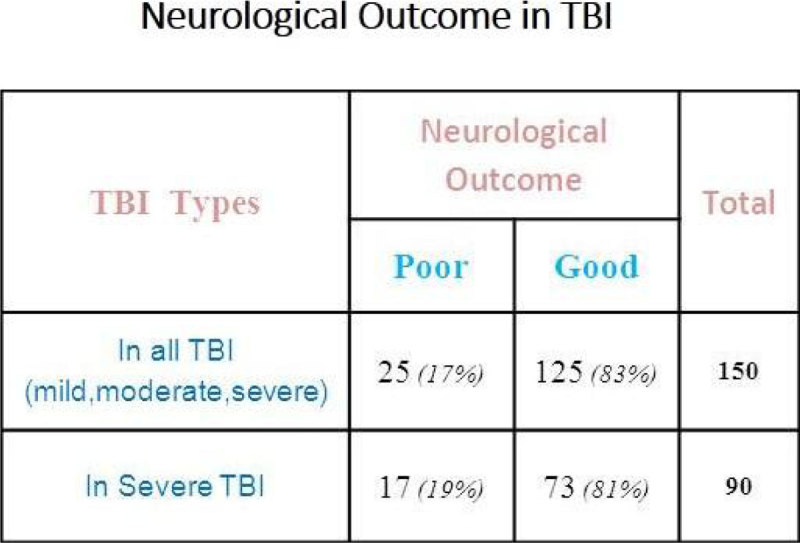
**[Neurological Outcome in TBI].**

**Figure 3:**
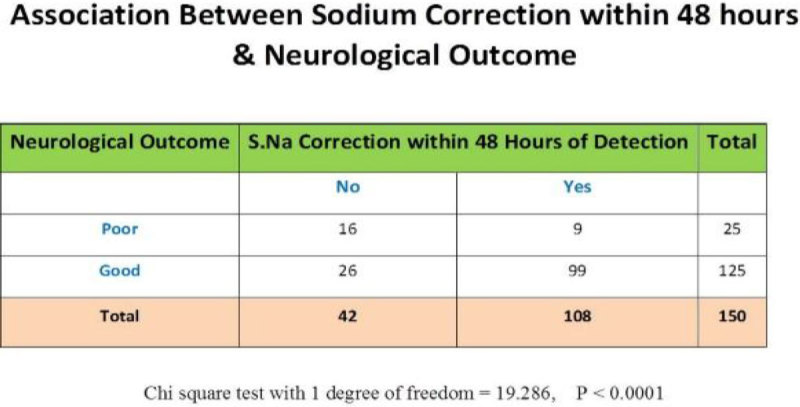
**[Early Correction of Sodium & Neurological Outcome].**

